# Deep-Learning-Based Semantic Labeling for 2D Mammography and Comparison of Complexity for Machine Learning Tasks

**DOI:** 10.1007/s10278-019-00244-w

**Published:** 2019-06-13

**Authors:** Paul H. Yi, Abigail Lin, Jinchi Wei, Alice C. Yu, Haris I. Sair, Ferdinand K. Hui, Gregory D. Hager, Susan C. Harvey

**Affiliations:** 1 The Russell H. Morgan Department of Radiology and Radiological Science, Johns Hopkins University School of Medicine, 601 N. Caroline St., Room 4223, Baltimore, MD 21287 USA; 2 Radiology Artificial Intelligence Lab (RAIL), Malone Center for Engineering in Healthcare, Johns Hopkins University Whiting School of Engineering, Baltimore, MD USA

**Keywords:** Deep learning, Semantic labeling, Mammography, Artificial intelligence, Breast tissue density

## Abstract

Machine learning has several potential uses in medical imaging for semantic labeling of images to improve radiologist workflow and to triage studies for review. The purpose of this study was to (1) develop deep convolutional neural networks (DCNNs) for automated classification of 2D mammography views, determination of breast laterality, and assessment and of breast tissue density; and (2) compare the performance of DCNNs on these tasks of varying complexity to each other. We obtained 3034 2D-mammographic images from the Digital Database for Screening Mammography, annotated with mammographic view, image laterality, and breast tissue density. These images were used to train a DCNN to classify images for these three tasks. The DCNN trained to classify mammographic view achieved receiver-operating-characteristic (ROC) area under the curve (AUC) of 1. The DCNN trained to classify breast image laterality initially misclassified right and left breasts (AUC 0.75); however, after discontinuing horizontal flips during data augmentation, AUC improved to 0.93 (*p* < 0.0001). Breast density classification proved more difficult, with the DCNN achieving 68% accuracy. Automated semantic labeling of 2D mammography is feasible using DCNNs and can be performed with small datasets. However, automated classification of differences in breast density is more difficult, likely requiring larger datasets.

## Introduction

A radiologist’s workflow depends on semantic labeling of digital images for tasks ranging from picture archiving and communication system (PACS) hanging protocols (how images are displayed on a monitor) to report generation. For example, semantic labeling of radiographic view facilitates the automated arrangement of images and comparison studies according to a designated hanging protocol, which provides time savings for a radiologist in every study he or she interprets. Further complexity is added to the workflow, however, when studies performed at outside hospitals are presented for second-opinion review with variable semantic labels for items such as study type and radiographic view. Although the Digital Imaging and Communications in Medicine (DICOM) imaging format stores metadata for semantic labels, its inclusion is inconsistent, can vary between equipment manufacturers, is frequently not in query-able format, and can be inaccurate [1]. As general labeling errors have been reported to be as high as 2.4% in plain radiographs [2], there is a need for quality assurance tools for accurate semantic labeling of medical imaging. Accordingly, an automated method for semantic labeling of medical imaging could help improve patient care and radiologist workflow, as well as facilitate curation of large imaging datasets for machine learning purposes [1]. This would be especially true for work which uses images from multiple different sites, which may have variable and/or inaccurate DICOM metadata labeling.

Deep learning has shown great promise in the automatic semantic labeling of medical images, such as classifying chest radiographs as frontal vs. lateral view [1] and laterality of ocular fundus photographs [3] with accuracies approaching 100%. These two studies [1, 3] utilized datasets of 150,000 chest radiographs (using standard data augmentation techniques) and 25,911 ocular fundus images, respectively; however, curating datasets with tens or hundreds of thousands of images may not be logistically feasible for many machine learning projects. Additionally, early work in pneumonia detection on chest radiographs [4] and accurate classification of chest vs. abdominal radiographs [5] suggests that for certain tasks, modest size image datasets on the scale of hundreds to thousands could result in deep learning systems (DLS) with high performance. Depending on the task, the accuracy of the DLS trained using moderate size datasets may be similar to those trained using large datasets with tens of thousands of images. At this time, it remains unclear how many images might be needed for medical imaging classification tasks of varying complexity.

Mammography is performed for the purpose of early breast cancer detection. One use of mammography is to screen asymptomatic women for breast cancer, which is recommended annually for all women of average risk over the age of 40 by the American College of Radiology [6]. Due to strict national regulations for DICOM labeling by the Mammography Quality Standards Act (MQSA) [7] and the resulting robust quality control, mammography serves as a potential model to explore the nuances of developing semantic labeling algorithms. A screening 2D mammographic examination consists of one craniocaudal (CC) view of the left and right breasts and a mediolateral oblique (MLO) view of each breast; all images have strict labeling requirements. Lessons learned from training mammography semantic labeling DLS with regard to the number of images needed for high-performing systems could be applied towards other modalities and more complex, but analogous, problems. Additionally, as mammographic semantic labeling tasks range from relatively simple tasks with fairly obvious differences between classes, such as mammographic view, to more complex ones with more subtle differences, such as breast density, challenges unique to these different tasks could also provide guidance for future DLS development.

The purpose of this study was to (1) develop deep convolutional neural networks (DCNNs) for automated classification of 2D mammography views, image laterality, and breast tissue density; and (2) compare the performance of DCNNs for each of these variably complex tasks to each other. We hypothesized that DCNNs would perform better for simpler classification tasks, such as 2D mammography views, than for more complex tasks, such as breast tissue density.

## Materials and Methods

All data used in this study were publicly available and de-identified, as described below. Our institutional review board (IRB) classified this study as non-human subjects research; accordingly, formal IRB review was not required per our institutional policies.

### Dataset

We utilized the Digital Database for Screening Mammography [8], which consists of 3034 2D de-identified digitized film mammographic images obtained in 2620 patients from 4 hospitals (Massachusetts General Hospital, Wake Forest, Sacred Heart, and Washington University in St. Louis). The images contain cases consisting of normal or benign examinations (i.e., with at least one mass proven to be benign by pathology, ultrasound, or imaging follow-up), as well as malignant (cancerous) findings on the examinations (with at least one pathology-proven cancerous mass); there are 1704 normal or benign examinations (56%) and 1130 malignant examinations (44%). Each image in this database was labeled with the following metadata, which were utilized for the 3 experiments in this study (described below): mammographic view (craniocaudal [CC] or mediolateral oblique [MLO]), breast laterality (right vs. left), and Breast Imaging Reporting and Data System (BI-RADS) breast density (4 categories: almost entirely fatty, scattered area of fibroglandular density, heterogeneously dense, and extremely dense). The distribution of these labels was spread relatively evenly, with the exception of breast density, which had a relative paucity of almost entirely fatty and extremely dense breasts (Table 1); this skewed distribution was expected, based on the average distribution of breast density in the female population [9].

**Table 1:** Mammography image labels and dataset distributions

	Total label nos. (3034)	Training (70%)	Validation (10%)	Testing (20%)
Mammographic view	CC: 1429 (47%)	CC: 1000	CC: 143	CC: 288
	MLO: 1605 (53%)	MLO: 1123	MLO: 161	MLO: 323
Laterality	Left: 1560 (51%)	Left: 1092	Left: 156	Left: 314
	Right: 1474 (49%)	Right: 1032	Right: 148	Right: 296
Breast density (BI-RADS)	A: 416 (14%)	A: 291	A: 42	A: 85
	B: 1182 (39%)	B: 827	B: 119	B: 238
	C: 928 (31%)	C:649	C: 93	C: 188
	D: 508 (16%)	D: 355	D: 51	D: 104

*CC* craniocaudal, *MLO* mediolateral oblique, *BI-RADS* Breast Imaging Reporting and Data System, *A* fatty, *B* scattered fibroglandular, *C* heterogeneously dense, *D* dense

### Image Processing and Computer Specifications

We saved all mammography images into portable network graphics (PNG) format and resized all images into a 256 × 256 matrix. Additionally, for the breast laterality dataset, we cropped out any laterality markers to prevent the DCNN from potentially learning to identify laterality markers or text, as opposed to the actual breast orientation.

For image processing, we utilized a computer running Linux with a Core i5 central processing unit (CPU) (Intel, Santa Clara, CA), 8 GB RAM, and a GeForce GTX 1050 graphics processing unit (GPU) (Nvidia Corporation, Santa Clara, CA). All programming was performed using the PyTorch deep learning framework (Version 0.3.1, https://pytorch.org). All DCNN development work was performed on remote computing servers with CPU and GPU nodes comprised of a dual socket 14-core 2.6 GHz CPU (Intel, Santa Clara, CA) with 128 GB RAM, and 2 Tesla K80 GPUs (Nvidia, Corporation, Santa Clara, CA), respectively.

### DCNN Development and Testing

We developed DCNN models for classification tasks of increasing complexity: 2D-mammographic view, image laterality, and breast tissue density. All tasks had 2 categories, except for breast density, which had 4 (Table 1). For each classification task, we created 3 datasets divided into training, validation, and testing sets, comprised of 70%, 10%, and 20% of images, respectively (Table 1), per standard DLS development methodology, whereby the majority of available images is used to train a DCNN (training phase), a smaller proportion is used to select the best-performing algorithm(s) (validation phase), and a separate holdout set of data to which the DCNN has never been exposed is used to test the performance of the best-performing DCNN(s) [5].

To develop our DCNNs, we applied transfer learning [1, 10] by utilizing the ResNet-50 [11] DCNN pretrained on ImageNet (http://www.image-net.org/). We redefined the last linear layer of the ResNet-50 DCNN to have 2 or 4 outputs instead of the default 1000 for the 3 FFMD image classification tasks (2 for 2D FFMD view and laterality; 4 for breast density). During training and validation of our DCNNs, all model parameters (initially configured to the pretrained ImageNet weights) were fine-tuned on the mammography images. The solver parameters we utilized for DCNN training were as follows: 49 epochs, stochastic gradient descent with a learning rate of 0.001, momentum of 0.9, and weight decay of 1 × 10^5^. During each training epoch, images were augmented on-the-fly via random rotations, cropping, and horizontal flipping.

We produced heatmaps for each DCNN using *class activation mapping* (CAM) [12] to visualize the parts of each image that were weighted most heavily by the DCNNs in making their classification decisions. In these heatmaps, red colors signify increasing importance of an image feature in the decision made by a DCNN.

### Statistical Analysis

We measured DCNN testing performance for binary classification tasks using receiver-operating characteristic (ROC) curves with area under the curve (AUC) generated. Optimal diagnostic thresholds were determined with the aid of the F1 score to calculate test sensitivity and specificity. For the 4-class breast density classification task, we calculated accuracy, sensitivity, and specificity of correctly classifying the density class. The DeLong non-parametric method [13] was used to compare AUCs between DCNNs [5, 10]. *P* values of < 0.05 were considered statistically significant.

## Results

**Fig. 1:**
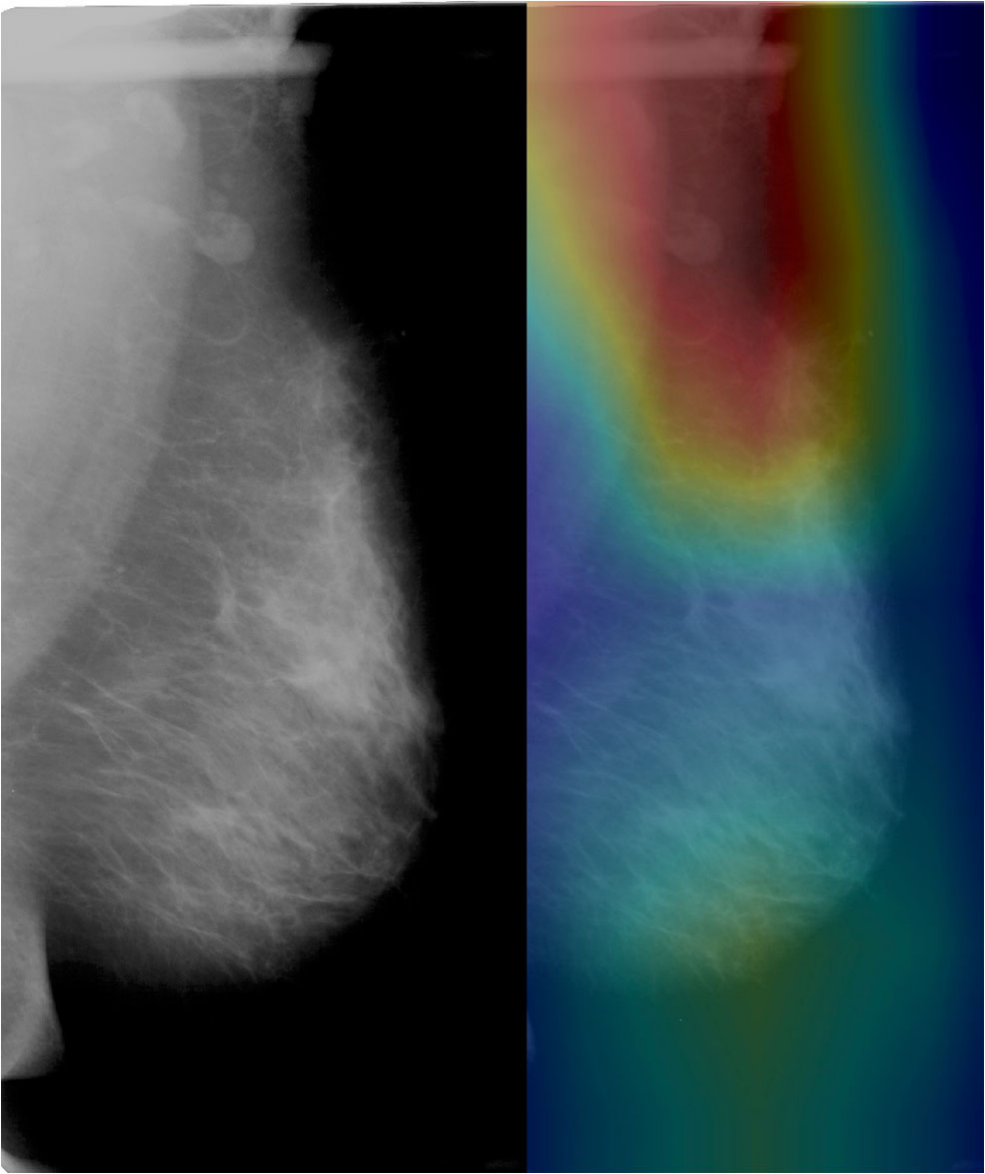
Heatmap of DCNN’s correct classification of MLO view shows emphasis of the superior interface between the pectoralis major muscle and breast tissue, consistent with features that a radiologist would utilize

The best-performing DCNN trained to classify CC vs MLO mammographic views (the simplest task) achieved an AUC of 1 with optimal sensitivity and specificity of 98% and 100%, respectively. CAM heatmaps demonstrated emphasis on the superior aspect of the imaged breast in classification of mammographic view, which corresponds to the pectoralis muscle and breast tissue for the MLO view (Fig. 1) and breast tissue alone for the CC view.

**Fig. 2:**
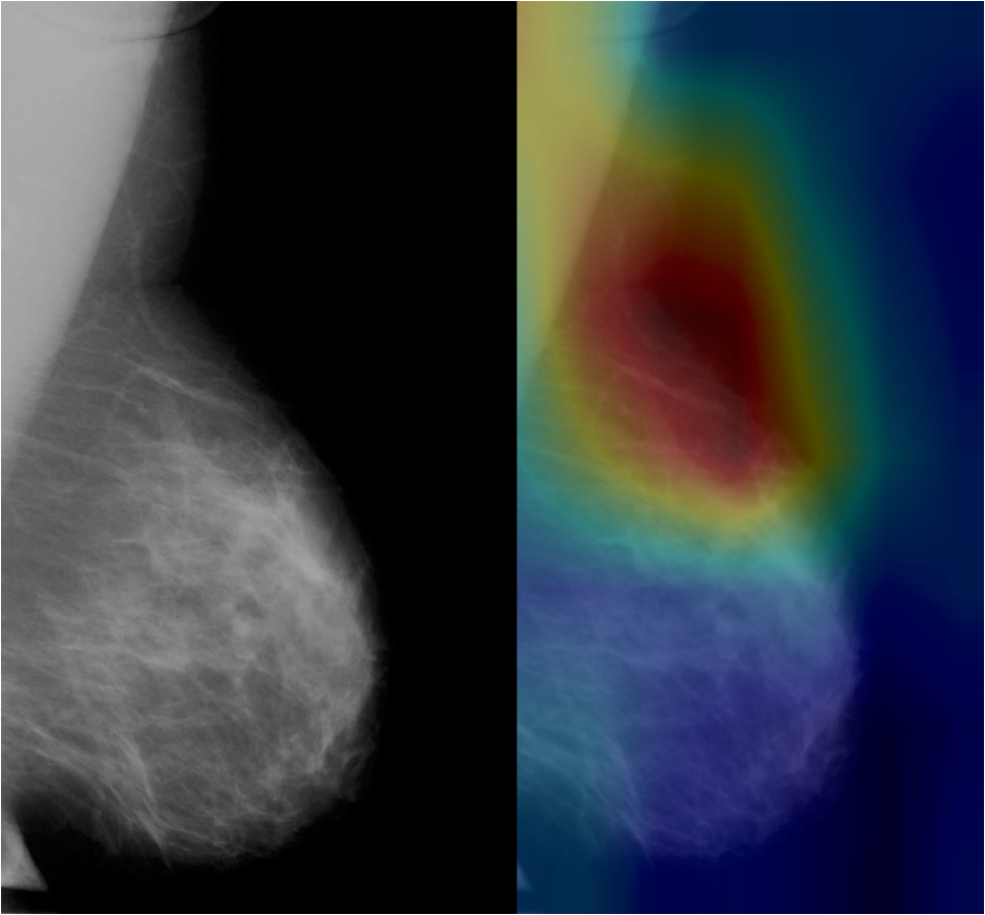
Heatmap of DCNN’s correct classification of left breast shows emphasis of the leftward-pointing breast convexity, consistent with features that a radiologist would utilize in classification

For the second, slightly more complex task of identifying breast laterality, the best-performing DCNN initially achieved AUC of 0.74 with optimal sensitivity and specificity of 83% and 51%, respectively. However, after discontinuing horizontal flips during data augmentation, the AUC improved significantly to 0.93 (*p* < 0.0001), with optimal sensitivity and specificity of 91% and 78%, respectively. CAM heatmaps demonstrated emphasis on the rightward or leftward-pointing breast convexities (Fig. 2).

**Fig. 3:**
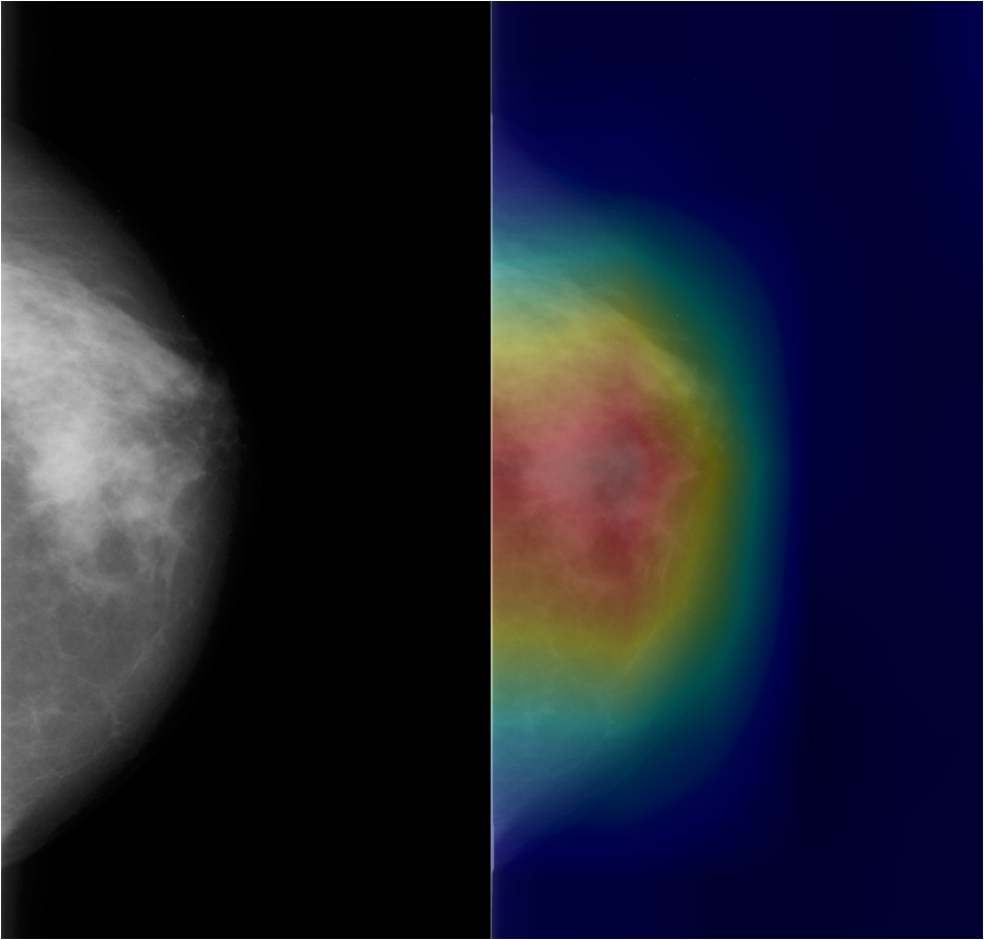
Heatmap of DCNN’s correct classification of dense breast tissue shows emphasis of the dense breast parenchyma, consistent with features that a radiologist would utilize in classification

The more complex task of classifying breast density into one of the 4 BI-RADS categories was not as successful, with accuracy of 68% and sensitivity and specificity of 90% and 53%, respectively. CAM heatmaps demonstrated consistent emphasis of the breast glandular tissue (Fig. 3) regardless of true breast density class or whether or not the DCNN correctly classified the breast density.

## Discussion

Deep learning has shown potential for automated semantic labeling of medical imaging [1, 3, 5, 14] for the purposes of improving patient care and radiologist workflow, and curating large datasets for machine learning purposes. In the present study, we developed 3 DCNNs for mammography image semantic labeling tasks of variable complexity using a moderate size dataset of 3034 images. We demonstrated higher performance in simpler tasks. Interestingly, we demonstrated higher performance for breast laterality classification when omitting horizontal flips during data augmentation, contrary to general machine learning principles.

The DCNNs trained in our study achieved AUC of 1 for distinguishing the CC and MLO mammographic views and 0.93 for breast laterality, despite the modest dataset size of 3034 images. Our findings are consistent with those reported previously by Rajkomar et al., who trained a DCNN to classify chest radiographs into frontal vs. lateral views, using 150,000 images, and achieved an AUC of 1 [1]. Similarly, Lakhani previously trained a DCNN to classify chest vs. abdominal radiographs (a similar task to determining radiographic view) with an AUC of 1, albeit with a smaller number of images for training and validation (90 images) [5]. As breast laterality proved more difficult for the DCNN to classify, increasing dataset size would likely improve performance; recent work in classification of ocular fundus image laterality achieved 99% DCNN accuracy when using 25,911 images [3]. Nevertheless, our findings suggest that high levels of performance can be achieved for relatively simple tasks with a modest training sample size.

The DCNNs in our study were less successful at the more difficult task of classifying breast tissue density, achieving 68% accuracy for classification into one of four categories. This decreased performance for tasks with increased subtleties between categories is consistent with human radiologist experience; generally speaking, mammographic view and laterality are more obvious to determine than breast density. Our findings are consistent with those demonstrated by Lakhani, who showed decreasing performance for tasks of increasing complexity (e.g., endotracheal tube presence vs. endotracheal tube positioning) [5]. Our findings suggest that datasets to train high-performing DCNNs for specific organ characteristics, such as breast tissue density, likely need to be larger than those used for pure semantic labeling. Indeed, recent studies have utilized larger datasets ranging from 22,000 to 41,479 images to train DCNNs for breast tissue density classification on mammography with performance as high as AUC of 0.94 [15, 16].

An interesting finding was that in the development of the breast laterality DCNNs, there was significantly higher performance when omitting horizontal flips during data augmentation. This finding may seem intuitive, as determination of breast laterality depends on horizontal orientation of the breast concavity (i.e., does it point towards the right or the left?). Unlike in imaging of other anatomic areas, right and left breasts are tremendously symmetric, which makes horizontal flips problematic. In contrast, other anatomic areas will have asymmetries, which makes such flipping less troublesome for appropriate image classification and laterality identification. For example, in the abdomen, the liver is a right-sided organ and the spleen is a left-sided organ, and so even in the presence of a horizontal flip, determining the right and left sides would be considerably easier than for a breast. Canonical machine learning theory holds that the more data augmentation performed, the better, ostensibly to increase the training image diversity and reduce the chances of overfitting. In contrast, our findings suggest that more data augmentation is not necessarily better; data augmentation should, therefore, be performed in a thoughtful manner, tailored to the task at hand. We emphasize, however, that our findings do not explain *why* omitting horizontal flipping improved DCNN performance. While it is logical that the horizontal flips would confuse the DCNN for laterality classification (as it would a human), it is unclear if this is definitely true for the DCNN. Future work could thus be directed to better understand the impact of these standard data augmentation techniques on network behavior.

As discussed above, we have demonstrated that DCNNs for simpler semantic labeling tasks achieve higher levels of performance than for more difficult tasks when given the same amount of data. However, we did not explore what is the lowest number adequate for training DCNNs for simpler semantic labeling tasks (i.e., how low can we go?). Prior work has shown that for relatively easy tasks, such as classifying radiographs into anatomic regions, DCNNs can be trained with high accuracy with as few as 90 training/validation images [5], but the optimum number is unclear for other tasks, such as imaging view or laterality. Future study in this area could facilitate the most efficient efforts to curate datasets for DLS training. Such information could help researchers in optimum allocation of resources for DCNN development; for example, if only 200 images are sufficient to classify radiographic view, then curating a dataset of 2000 would be unnecessary and inefficient.

Our study had several limitations. First, our dataset was small, consisting of 3034 images, which limits the ability of DCNNs’ diagnostic performance. Importantly related to this limitation, one of our goals was to explore differential performance ability given a modest dataset in order to hypothesize about the size of the dataset for certain classification tasks for DLS development. Second, semantic labeling of mammography is generally not a prominent clinical problem in the USA, due to federally regulated MQSA guidelines and the resultant stringent quality control (which includes mandates to include such information). In fact, we used this mandate and the labels as a way to ensure an accurately annotated dataset for our experiments, as one goal was to gain insight into image dataset size for DCNN training. Nevertheless, semantic labeling DCNNs for mammography could be useful in settings outside of the USA, where mandates such as the MQSA guidelines do not exist; particularly, if one wanted to pool data from multiple sites and countries for DLS development, such semantic labeling tools could be useful. Third, we utilized only one DCNN architecture in our study and did not test the performance or utility of multiple DCNNs, either in isolation or in combination, as has been done in prior studies [5, 10]; a different DCNN architecture or combinations could possibly improve performance.

In conclusion, automated semantic labeling of 2D mammography is feasible using DCNNs and small image datasets. However, automated classification of more subtle differences such as breast density is a more difficult task, likely requiring larger datasets, although optimal data size is indeterminate. While previously, data augmentation has been shown to increase DCNN performance, certain augmentation techniques may actually be detrimental depending on the DCNN’s goal task and careful consideration of how and when to use these techniques is an important finding in our work. Practically, in our team’s development of laterality-classifying DCNNs, we no longer implement horizontal flipping, as this results in worse classification ability.
